# Chemical Composition, Physicochemical Properties, and Antimicrobial Activity of Stingless Bee Geopropolis from the Baturité Massif, Northeastern Brazil

**DOI:** 10.1021/acsomega.6c00112

**Published:** 2026-03-26

**Authors:** João V. L. Teixeira, Francisco I. S. Martins, Franciany C. Carmo, Isnara S. Holanda, Marcelo C. Cavalcante, José D. B. Costa Filho, Lourena M. Veríssimo, Dayanne L. Porto, Cícero F. S. Aragão, Wilma R. V. Rocha, Jamerson F. Oliveira, Luanne E. Nunes, Marcelo V. P. Amorim

**Affiliations:** † Institute of Health Sciences, University of International Integration of the Afro-Brazilian Lusophony (UNILAB), Redenção, Ceará 62790-000, Brazil; ‡ Postgraduate Program in Biotechnology (RENORBIO), State University of Ceará, Fortaleza, Ceará 60740-000, Brazil; § Institute of Rural Development, University of International Integration of the Afro-Brazilian Lusophony (UNILAB), Redenção, Ceará 62790-000, Brazil; ∥ Center for Food and Drug Research (NUPLAM), 28123 Federal University of Rio Grande do Norte (UFRN), Natal, Rio Grande do Norte 59078-970, Brazil; ⊥ Department of Pharmacy, Federal University of Rio Grande do Norte (UFRN), Natal, Rio Grande do Norte 59078-970, Brazil; # Postgraduate Program in Pharmaceutical Sciences, State University of Paraíba, Campina Grande, Paraíba 58429-500, Brazil

## Abstract

Propolis is a resinous material collected by bees and is rich in phenolic and terpenoid compounds with antimicrobial and antioxidant properties. In Brazil, propolis produced by stingless bees incorporates soil-derived materials, forming geopropolis with distinct physicochemical characteristics. This study investigated the chemical profile, physicochemical properties, and antimicrobial potential of geopropolis produced by *Scaptotrigona aff*. *depilis* and *Frieseomelitta varia* from Ceará, Brazil, using integrated in vitro and in silico approaches. Marked interspecific differences were observed, with higher wax and mechanical impurity contents in *S. depilis*, whereas *F. varia* presented a higher resin fraction. Both samples contained phenolics and flavonoids, and phytochemical screening revealed tannins, coumarins, alkaloids, and steroids. GC–MS identified triterpenes and sterols as major constituents in *S. depilis*, while diterpenes predominated in *F. varia*. Antimicrobial assays demonstrated inhibitory activity against tested microorganisms, with minimum inhibitory concentration (MIC) values ranging from 32 to 4096 μg/mL, with the strongest activity observed for geopropolis produced by *F. varia*. Molecular docking showed strong binding affinities toward β-lactamase KPC-2 and dihydropteroate synthase. These results highlight geopropolis from stingless bees as a promising natural source of bioactive compounds with potential activity against antibiotic-resistant pathogens.

## Graphical abstract

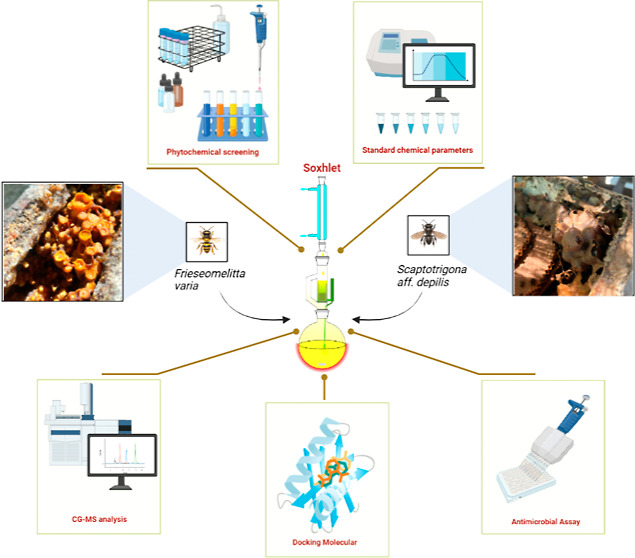

## Introduction

Propolis is a product made by bees from a mixture of plant tissue fragments, bee wax, saliva, and plant resins, which serves to protect the hive both physically and microbiologically.^1–3^ Phenolic compounds and terpenoids are the main constituents of propolis and are responsible for its antimicrobial, antioxidant, and anti-inflammatory properties.^4^

In Brazil, most research on propolis has focused on the exotic honeybee *Apis mellifera*, whose propolis primarily consists of resin (50%), wax (30%), essential and aromatics oils (10%), bee pollen (5%), and other substances (5%). Stingless bees, a eusocial group that plays an important role in pollination, also produce a variety of propolis, popularly known as geopropolis.^5^ It consists of a mixture of resin, wax, and soil with distinctive physicochemical characteristics. As with propolis, geopropolis is used to line the entrance of the hive and seal holes on the hive nest.^6^ In the Caatinga of northeastern Brazil, a region rich in stingless bee diversity, at least forty-nine species have been recorded.^7^ The bees are called “stingless” because their sting is atrophied, and they are also known as indigenous bees.^8^

In Brazil, geopropolis has been used for the treatment of several conditions such as respiratory diseases and dermatoses and has demonstrated antioxidant, anticancer, anti-inflammatory, and antimicrobial activities.^9^ Furthermore, the study by Pereira et al. showed that geopropolis samples have cytotoxic activity against several human cancer cell lines.^10^

*Scaptotrigona aff.* *depilis* and *Frieseomelitta varia* are native stingless bee species of the Caatinga biome, widely distributed and commonly maintained in managed meliponaries. These species actively collect and store significant amounts of geopropolis, making them key candidates for studies of this valuable natural product. However, because of their presence in the Caatinga ecosystem of the Baturité Massif region, Ceará, Brazil, the chemical composition and biological properties of their geopropolis remain largely unexplored.

In this context, this study provides a comparative investigation of geopropolis produced by *S. depilis* and *F. varia* collected in the Baturité Massif. The novelty of this work lies in the integration of complementary analytical approaches, including physicochemical characterization, chemical profiling by gas chromatography–mass spectrometry (GC–MS), antimicrobial evaluation through minimum inhibitory concentration (MIC) and minimum bactericidal concentration (MBC) assays, and molecular docking analysis to explore potential interactions between identified compounds and microbial targets. By combining chemical, microbiological, and in silico approaches, this study aims to provide a broader understanding of the relationship between the chemical composition of geopropolis and its antimicrobial potential, contributing to the characterization of geopropolis from a region that remains relatively underexplored.

## Materials and Methods

### Propolis Samples

Geopropolis samples of *S. aff.* *depilis* and *F. varia* were collected in March 2024 at the Baobá Meliponary, University of International Integration of Afro-Brazilian Lusophony (UNILAB), Acarape, Ceará, Brazil (4°13′06.1″ S, 38°42′46.9″ W). Access to the genetic heritage associated with the biological material was registered in the Brazilian National System for the Management of Genetic Heritage and Associated Traditional Knowledge (SISGEN; registration no. A9AF70E). Approximately 20 g of geopropolis from three independent colonies of each species was collected by scraping the material that accumulated between the hive and its cover. The samples were pooled prior to any treatment, manually cleaned to remove visible impurities, transferred to plastic tubes, and stored at −20 °C. After being frozen, the geopropolis was pulverized using a mortar and pestle.

### Physical-Chemistry Analysis

The physicochemical characterization of the powdered samples was conducted following the methodology proposed by Woisky and Salatino, with modifications.^11^ Moisture content was determined by drying 3 g of each sample at 105 °C for 2 h until constant weight, while ash content was obtained by calcination at 600 ± 25 °C until complete whitening of the residue. Ethanol extractions were performed in triplicate using 5 g of the sample in a Soxhlet apparatus for 8 h, followed by overnight storage at −20 °C. The wax fraction was isolated by centrifugation of the ethanolic extract at 4500 rpm for 5 min at −20 °C, and the precipitate was dried at 105 ± 5 °C to constant weight. The mechanical mixture was obtained from the Soxhlet residue after ethanol extraction and subjected to drying under the same conditions. Finally, resin content was quantified from the wax-free ethanolic extract—*F. varia* (WFE-FV) and *S. depilis* (WFE-SD)—by heating 5 mL aliquots at 105 °C ± 5 °C for 2 h until constant weight. For each determination, the initial sample mass was corrected by subtracting the previously determined moisture content. Thus, calculations were based on the corresponding dry mass of each analyzed fraction, ensuring accuracy and consistency across all parameters. All analyses were performed in triplicate, and results were expressed as percentages by weight (% w/w).

### Phytochemical Screening

For the identification of the different groups of secondary metabolites present in the WFE-FV and WFE-SD, the techniques and procedure described by Harborne were used.^12^

### Standard Chemical Parameters

Total phenolic content of WFE-FV and WFE-SD was estimated by the Folin–Ciocalteu method, with some modifications.^13^ Absorbance at 760 nm was measured after incubation for 30 min at room temperature. Quercetin (0–8 mg/L) was used for the standard calibration curve. The results were expressed as milligrams of Quercetin equivalent (QE)/g of dry weight and calculated as mean value ±SD (*n* = 3). Total flavonoid content was determined according to Asem et al., with some modifications.^13^ The absorbances of WFE-FV and WFE-SD were measured at 425 nm after 30 min of incubation at room temperature. Quercetin (0–12 mg/L) was used for the standard calibration curve. The results were expressed as mg Quercetin equivalent (QE)/g dry weight and calculated as mean value ±SD (*n* = 3).

### CG-MS Analysis

The WFE-FV and WFE-SD samples were analyzed using gas chromatography–mass spectrometry (GC-MS) quadrupole analyzer equipment (Agilent—Wilmington, USA) equipped with an HP-5 MS capillary (30 m length × 0.25 mm internal diameter and 0.25 μm film thickness). The carrier gas was helium at a flow rate of 1 mL/min. The mass spectrometer was operated by using electron impact at 70 eV, with a solvent delay of 5 min. The scan was in SCAN mode in the range of 40–550 *m*/*z* with a sampling rate of 5.42 scans/s. The transfer line, ion source, and quadrupole used the following temperatures: 280 °C, 230 °C, and 150 °C, respectively. Data acquisition was carried out using the OpenLab CDS software (version 2.5). *F. varia*: WFE-FV samples (1 μL) were injected with an auto sampler in split ratio 2:1 at an injector temperature of 280 °C. The oven was initially at 150 °C for 5 min, increased at a rate of 5 °C/min to 230 °C, storage time of 10 min, and then increased at the same rate to 300 °C. The total GC–MS running time was 45 min. *S. aff.* *depilis*: WFE-SD samples (1 μL) were injected with an autosampler in split ratio 10:1 at an injector temperature of 280 °C. The oven was initially at 175 °C for 5 min, increased at a rate of 5 °C/min to 290 °C, stored for 10 min, and then increased at rate of 1 °C/min to 300 °C, which was maintained for 5 min. The total GC–MS running time was 53 min. Using the National Institute of Standards and Technology (NIST 2017 library) database, the mass spectrum was used to identify the name, molecular weight, and structure of the components of both samples. Compounds were considered when the match score exceeded 800.

### Molecular Docking

The in silico pharmacodynamic antimicrobial activity was investigated using the major phytoconstituents identified by GC–MS (Table 5), targeting the enzymes dihydropteroate synthase (DHPS; PDB ID: 5U0 V) and KPC-2 (PDB ID: 5UJ4). DHPS was selected because it is an essential enzyme conserved in the folate biosynthesis pathway in a wide range of bacteria, being present in both Gram-positive and Gram-negative microorganisms. The bacterial species evaluated in this study are known to encode DHPS, supporting the relevance of this enzyme as a molecular target. DHPS inhibition compromises bacterial growth and survival.^14^

KPC-2, a class A serine β-lactamase, was included due to its crucial role in β-lactam resistance, particularly *Enterobacterales*. This enzyme is especially relevant for *Klebsiella pneumoniae*, a strain associated with the production of extended-spectrum β-lactamase and carbapenemase, and represents an important mechanism of resistance that is clinically widespread in Brazil. The active site of KPC-2 allows the hydrolysis of most β-lactam antibiotics through the formation of a covalent acyl–enzyme complex, contributing to high levels of antimicrobial resistance.^15^

The three-dimensional structures of the proteins were retrieved from the Protein Data Bank (https://www.rcsb.org/structure). The molecular structures of the phytoconstituents identified in *F. varia*: Kaur-16-en-18-ol (4α), Kaur-16-en-18-al (4α), Dehydroabietic acid, and Kauren-19-oic acid; and in *S. depilis*: β-Amyrone, β-Amyrin, Lanosterin, Lup-20(29)-en-3-one, Lupeol, and Lupeol acetate; were obtained in two-dimensional (2D) format from PubChem (https://pubchem.ncbi.nlm.nih.gov) and ChemSpider (https://www.chemspider.com) databases. They were then converted to three-dimensional (3D) format, protonated, adjusted to physiological pH (7.4), subjected to energy minimization, and saved in a PDB format. In AutoDockTools, Gasteiger charges were assigned, rotatable bonds were defined, and the files were converted to the PDBQT format. The same procedure was applied to the reference inhibitors of each target protein. Protein preparation involved the removal of water molecules, the addition of polar hydrogens, and the exclusion of cocrystallized ligands. The docking regions were defined based on the active sites of the enzymes, using the following coordinates: *X* = 17.519; *Y* = −3.354; *Z* = 49.223 for DHPS and *X* = 21.885; *Y* = 14.678; *Z* = 13.433 for KPC-2. The methodological validation was carried out through the redocking of cocrystallized ligands using AutoDock Vina.^16^ The accuracy of the method was verified based on RMSD values determined in Discovery Studio. After validation, molecular docking simulations were performed in AutoDock Vina, during which the binding energies (kilocalories per mole) were recorded. The lowest-energy conformations, corresponding to interactions within the active sites of the proteins, were selected for detailed analysis.

### Antimicrobial Assay

The Minimum Inhibitory Concentration (MIC) was determined using the broth microdilution method in 96-well microplates, according to CLSI guidelines.^17^ WFE-SD and WFE-FV were tested at concentrations ranging from 2048 to 0.5 μg/mL against the standard strains *Staphylococcus aureus* ATCC 25923, *S. aureus* ATCC 33591, *Escherichia coli* ATCC 25922, *Pseudomonas aeruginosa* ATCC 27853, *P. aeruginosa* PAO1, and *K. pneumoniae* ATCC 70063. The plates were incubated at 37 °C for 24 h. A control well containing the diluent (0.85% NaCl/96 °GL ethanol, 8:2, v/v) was included to exclude any antimicrobial effect attributable to the solvent system. Tetracycline and ciprofloxacin were used as reference antimicrobial agents. After incubation, MIC values were assessed by the addition of resazurin to all wells, followed by a second incubation at 37 °C for 3 h. The appearance of a blue color was interpreted as an indication of bacterial growth inhibition. The Minimum Bactericidal Concentration (MBC) was determined from wells that showed no visible bacterial growth. Aliquots of 10 μL were plated on Mueller–Hinton agar and incubated at 37 °C for 24 h for CFU enumeration. The MBC was defined as the lowest extract concentration capable of killing 99.99% of the bacterial population. The analyses were performed in triplicate, and the results were expressed as the mean.

### Statistical Analysis

Comparisons between groups were performed using the Prism 9 GraphPad Software with Student’s *t*-test after confirming the assumptions of normal distribution and homogeneity of variances. All experiments were performed in triplicate (*n* = 3), and the results are expressed as the mean ± standard deviation. The results were considered significant when *p* <0.05.

## Results/Discussion

The present study provides valuable insights into the chemical composition and biological activities of *S. depilis* and *F. varia* geopropolis. These findings may contribute to discussions regarding their potential applications in medicine and healthcare. Significant variations were detected in the quantitative and qualitative results of the physicochemical and biological analyses.

Measurements of humidity, ash, wax, and mechanical mixtures in the raw geopropolis as well as resin content and total phenolic and flavonoid contents of WFE-SD and WFE-FV are presented in Table 1.

**Table 1:** Physicochemical Parameters and Total Phenolic and Flavonoid Contents of Geopropolis Samples (WFE-SD and WFE-FV)^a^

crude geopropolis experiment	*S. depilis*	*F. varia*
humidity (% w/w)	6.9 ± 0.5^a^	4.2 ± 0.4^b^
ash (% w/w)	2.9 ± 0.9^a^	3.0 ± 0.3^a^
wax (% w/w)	13.7 ± 0.9^a^	4.7 ± 0.6^b^
mechanical mixtures (% w/w)	31.6 ± 1.6^a^	9.3 ± 0.4^b^

WFE experiment	WFE-SD	WFE-FV
resin (% w/w)	55.3 ± 2.3^a^	80.0 ± 3.8^b^
total phenolic (mg quercetin equivalent (QE)/g dry weight)	33.0 ± 0.1^a^	21.1 ± 0.1^b^
total flavonoid (mg quercetin equivalent (QE)/g dry weight)	7.2 ± 0.1^a^	5.0 ± 0.1^b^

a Significant differences (*p* <0.05) between groups were identified by Student’s *t*-test, as indicated by different letters assigned to each group within the same line.

The humidity content exhibited a statistically significant difference (*p* = 0.04) and ash tests demonstrated similar results when both samples were compared (*p* = 0.06). In geopropolis, soil is incorporated into its composition, which may include water particles. These colloidal structures exhibit high water retention capacity and are therefore highly responsive to variations in ambient humidity.^18^

The differences observed in the wax (*p* = 0.02), mechanical mixture (*p* = 0.03), and resin contents (*p* = 0.02) between the analyzed species reinforce the complex and variable nature of the geopropolis. The higher levels of wax and mechanical mixtures found in *S. depilis* suggest a more intense incorporation of lipid secretions and solid particles during the collection and elaboration processes. According to Cardinal et al., wax does not contain the main phenolic compounds responsible for the biological activity of propolis; therefore, higher wax contents may be associated with lower concentrations of resinous constituents and, consequently, a product with reduced bioactive potential.^19^ Thus, the lower wax content and higher resin percentage observed in *F. varia* indicate a geopropolis with a greater chemical potential and, possibly, higher pharmacological relevance.

Variations in wax and particulate matter contents may also reflect environmental and behavioral differences between the producing species. According to the authors, wax levels may vary depending on the region and climatic conditions, being influenced by the need for hive sealing during periods of higher humidity or cold. Therefore, the greater proportion of mechanical mixtures in *S. depilis* may be related both to the collection environment and to the characteristic constructive behavior of the species, which tends to produce a denser and more structurally oriented geopropolis.^19^

Geopropolis differs from conventional propolis because its formation involves the incorporation of soil or clay particles, resulting in a naturally higher mineral content. This mineral fraction has been explored as a potential traceability indicator since it may reflect the geochemical characteristics of the soil from the region where the geopropolis is produced. Furthermore, it may contribute nutritionally as a source of essential elements, including Ca, Cu, Fe, K, Mg, Mn, P, and Zn. Therefore, elevated levels of insoluble or mineral fractions in geopropolis should not necessarily be interpreted as impurities but rather as intrinsic characteristics of this material produced by stingless bees of *Meliponini*.^20^

Villacrés-Granda et al., in a study evaluating the mineral composition of stingless bee honey from Ecuador, reported that the concentrations of Cu, Pb, Ni, Mn, Zn, and Fe were below the limits of quantification, whereas Al was detected at low levels with an average concentration of 0.06 mg/g of honey. These findings suggest that, although several trace elements may occur at very low concentrations, the mineral fraction is a natural component of bee-derived products.^21^

In our study, we determined total phenolic and flavonoid contents by spectrometric methods and we found values between 33.0 ± 0.1 mg QE/g and 7.2 ± 0.1 mg QE/g, respectively, for *S. depilis* and 21.1 ± 0.1 mg QE/g and 5.0 ± 0.1 mg QE/g, respectively, for *F. varia*. The total phenolic (*p* = 0.03) and flavonoid (*p* = 0.04) contents exhibited a statistically significant difference when both samples were compared. In a study conducted by Bergamini et al., propolis of *Tetragona clavipes* exhibited a concentration of polyphenol content of 76.68 ± 1.74 mg gallic acid/g and flavonoid content of 23.97 ± 7.38 mg QE/g, during a rainy season, and a polyphenol content of 26.11 ± 1.04 mg gallic acid/g and flavonoid content of 22.82 ± 4.33 mg QE/g, during a dry season.^22^ Similar results were observed by Pazin et al., with a polyphenol content of 12.5 ± 0.3 mg gallic acid/g and flavonoid content of 1.8 ± 0.1 mg QE/g. Those results compared to our study may be attributed to the variations in the flowering period of plant species during different seasons.^23^ The low values can be explained by Soares et al., who reported in their study that variation in the flavonoid content of propolis is a well-established fact, since several factors influence its composition, such as the collection period, bee species, and the plant source involved.^22,24^ Indeed, phenolic groups are responsible for part of the therapeutic activity of propolis; however, the amount of these compounds is not directly associated with a higher or lower efficacy, given that this product contains other constituents that are equally or even more relevant to the expected biological activity.

### Phytochemical Screening

The phytochemical analysis of geopropolis extracts revealed the presence of tannins, flavonoids, coumarins, alkaloids, and steroidal compounds (Table 2), corroborating the literature data on the composition of products produced by stingless bees. Several studies have shown that species of the genus *Scaptotrigona* produce geopropolis and other bee-derived products rich in secondary metabolites, especially flavonoids, phenolic acids, terpenoids, and alkaloids, which are directly associated with the antioxidant, antimicrobial, and anti-inflammatory properties of these products.^25^ The authors also note that the absence of saponins in the analyzed samples is consistent with previous reports, as these compounds are rarely found in bee-derived propolis. Thus, the results reinforce that the chemical composition of geopropolis from *S. depilis* and *F. varia* follows the typical phytochemical profile observed in meliponines, characterized by the predominance of plant-derived phenolic compounds and terpenoids responsible for their range of biological activities.

**Table 2:** Phytochemical Screening of WFE-SD and WFE-FV

secondary metabolites	test	WFE-SD	WFE-FV
tannin	ferric chloride	+	+
flavonoid	Shinoda	+	+
	aluminum chloride	+	+
	Pew	+	+
	Taubouk	-	+
coumarin	fluorescence in alkaline medium	+	+
alkaloids	Dragendorff	+	+
	Mayer	+	+
steroids and triterpenes	Liebermann-Burchard	+ (green)	+ (green)
saponins	foam	–	–

The chemical profile of Meliponini propolis is predominantly defined by a diverse array of bioactive secondary metabolites—notably phenolic acids, flavonoids, and terpenes—whose quantitative and qualitative distributions are strictly governed by local botanical resources and species-specific foraging behaviors. These phytochemical constituents underpin the significant pharmacological potential of the material, exhibiting robust antioxidant, antimicrobial, and immunomodulatory activities that ensure the metabolic and hygienic stability of the colony.^26^

### CG-MS Analysis

The GC–MS analysis of WFE-SD and WFE-FV is presented in Figure 1 and Table 3. The major compounds were determined based on the highest intensity peaks in the analysis, indicating their significant presence in the sample.

**Figure 1:**
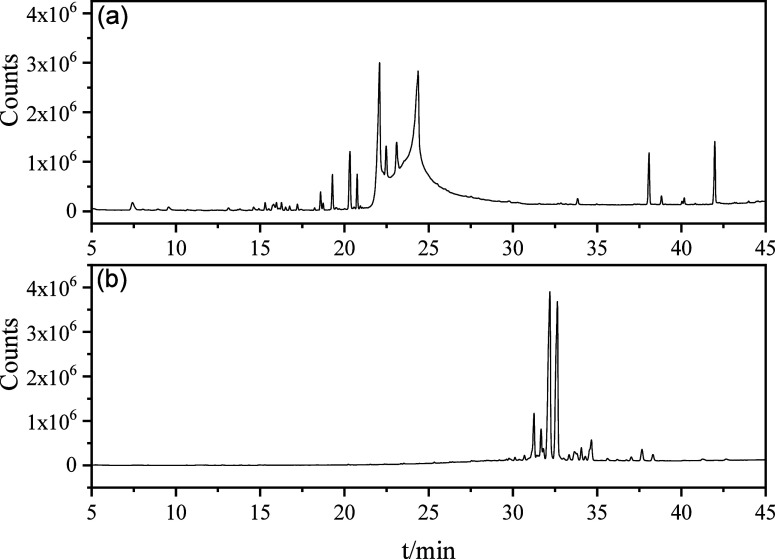
GC–MS chromatogram of WFE-SD (a) and WFE-FV (b).

**Table 3:** Compounds Identified in WFE-SD and WFE-FV Using GC–MS Analysis

WFE	compound	RT (min)	exact mass (g/mol)	molecular formula	relative abundances (%)
SD	Stigmasterol	30.12	412.69	C_29_H_48_O	0.34
	β-Amyrone	31.25	424.71	C_30_H_48_O	6.71
	β-Amyrin	31.67	426.7	C_30_H_50_O	3.71
	Lanosterin	31.80	426.72	C_30_H_50_O	1.19
	Lup-20(29)-en-3-one	32.19	424.71	C_30_H_48_O	36.52
	Lupeol	32.63	426.72	C_30_H_50_O	32.50
	24-methylenecycloartan-3-one	33.33	438.74	C_31_H_50_O	0.73
	Lupeol acetate	34.65	468.76	C_32_H_52_O_2_	4.21
FV	Spathulenol	7.42	220.35	C_15_H_24_O	0.92
	4(15),5,10(14)-germacratrien-1-ol	8.93	220.35	C_15_H_24_O	0.15
	Hibaene	14.61	272.47	C_20_H_32_	0.18
	Kaur-16-en-18-al, (4α)-	15.96	286.45	C_20_H_30_O	0.39
	Androst-2,16-diene	16.12	256.4	C_19_H_28_	0.14
	(−)-13-Epimanoyl oxide	16.26	290.48	C_20_H_34_O	0.38
	5α-Androstane-3β,17β-diol	16.51	292.46	C_19_H_32_O_2_	0.16
	Kaur-16-ene	16.75	272.47	C_20_H_32_	0.20
	Kaur-16-en-18-ol, (4α)-	20.32	288.47	C_20_H_32_O	3.42
	Dehydroabietic acid	22.08	300.4	C_20_H_28_O_2_	19.13
	15-Acetoxykaur-16-em-18-oic acid	22.48	360.49	C_22_H_32_O_4_	6.63
	Kauren-19-oic acid	24.37	302.45	C_20_H_30_O_2_	44.10
	Kauren-16-en-18-oic acid, 15-(angeloxy)-methylester, (4α,1)	38.81	415.58	C_26_H_38_O_4_	0.41

For *S. depilis*, the main compounds detected were stigmasterol, β-amyrin, β-amyrin, lanosterol, lup-20(29)-en-3-one, lupeol, 24-methylenecycloartan-3-one, and lupeol acetate. These results are consistent with previous GC–MS studies on *S. depilis*, which reported the presence of sterols and triterpenes such as β-sitosterol, β-amyrin, α-amyrin, and β-amyrin acetate, tocopherol, and sesquiterpenes including cyclosativene, α-duprezianene, α-guaiene, and germacrene A.^25^

For *F. varia*, the main compounds identified were spathulenol, 4(15),5,10(14)-germacratrien-1-ol, hibaene, kaur-16-en-18-al (4α), androst-2,16-diene, (−)-13-epimanoyl oxide, 5α-androstane-3β,17β-diol, kaur-16-ene, kaur-16-en-18-ol (4α), dehydroabietic acid, 15-acetoxykaur-16-en-18-oic acid, kauren-19-oic acid, and kauren-16-en-18-oic acid 15-(angeloxy)-methyl ester (4α,1). In the literature, studies of stingless bee propolis, particularly from the genus *Frieseomelitta*, are scarce. Available data indicate high variability in composition, with no single component being consistently present across all samples. Compounds such as α-cubebene, γ-yalangene, α-copaene, α-gurjunene, *E*-β-farnesene, and germacrene D have been previously reported for this genus, which partially agrees with our findings.^27^ These results reinforce that the chemical composition of the geopropolis can vary even within the same bee species.

The predominant compounds identified in *S. depilis* were lup-20(29)-en-3-one (36.52%) and lupeol (32.50%). In *F. varia*, the major constituents were kauren-19-oic acid (44.10%) and dehydroabietic acid (19.13%).

Lupeol is a triterpene widely described in the literature for its various biological activities, including antiprotozoal, antimicrobial, anti-inflammatory, antioxidant, antidiabetic, antitumor, and chemopreventive and healing effects. In contrast, there are no consistent reports of significant biological activity for lup-20(29)-en-3-one.^28^

Kaurenoic acid is a diterpenoid known for its anti-inflammatory and antimicrobial properties. However, its application as a functional ingredient is still limited due to issues related to the sustainability of extraction from plant sources, in addition to its low solubility in water.^29^

In turn, dehydroabietic acid is a tricyclic diterpenoid resin acid whose derivatives have been associated with a wide range of biological activities of medicinal and agricultural interest, including anticancer, antibacterial, antiviral, antiulcer, insecticidal, and herbicidal activities.^30^

### Molecular Docking

For the molecular docking studies, two biologically relevant target enzymes were selected: dihydropteroate synthase (DHPS), which plays a key role in folate biosynthesis, an essential process for bacterial survival, and KPC-2 β-lactamase, which is an enzyme responsible for carbapenem resistance. The latter represents one of the major global public health concerns due to the increasing resistance of these pathogens to most β-lactam antibiotics. In this context, bioinformatics tools were employed as a strategic approach for the preliminary screening of compounds with a potential antibacterial activity.

Prior to the docking simulations with the secondary metabolites, the native ligands of each enzyme were removed and subjected to structural optimization. The optimized ligands were then redocked into their respective active sites to validate the docking protocol. The resulting structural superpositions yielded RMSD values below 2 Å, demonstrating the high accuracy and reliability of the adopted methodology. According to Castro-Alvarez, Costa, and Vilarrasa, RMSD values ≤2 Å are considered appropriate for validating molecular docking procedures (Figure 2).^31^

**Figure 2:**
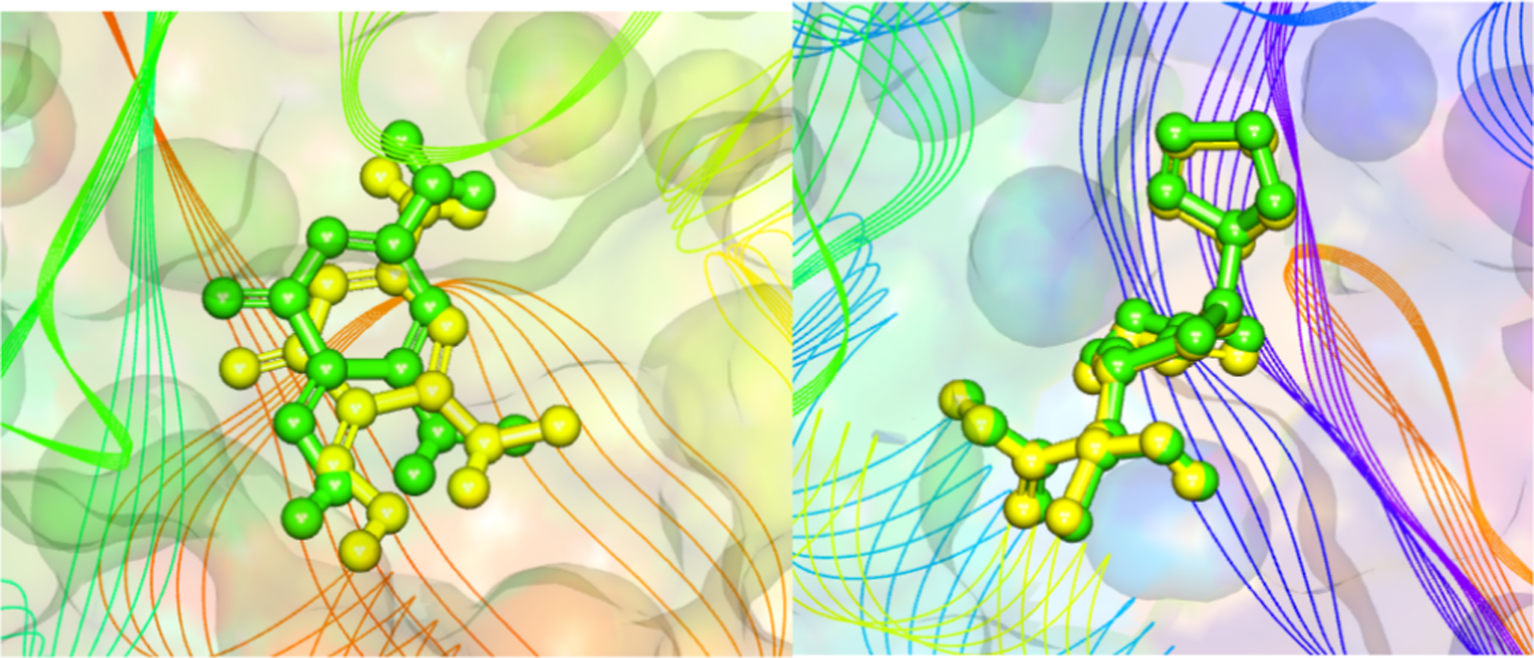
Validation of the docking protocol by overlapping the ligand (green) and after redocking (yellow) of dihydropteroate synthase (DHPS; RMSD = 0.9590 Å) and KPC-2 β-lactamase (RMSD = 0.270 Å), respectively.

The molecular docking results indicated that the main phytoconstituents identified in the extracts of *S. depilis* (SD) and *F. varia* (FV) exhibited distinct patterns of affinity and interaction toward the enzymes dihydropteroate synthase (DHPS, Figure 3) and KPC-2 (Figure 4) and are presented in Table 4. Binding energies ranged from −6.5 to −10.2 kcal/mol, with more negative (and thus more favorable) values observed for compounds from *S. depilis*, particularly against KPC-2. These values represent the theoretical in silico estimate of the binding affinities of the evaluated compounds for the selected targets. However, to confirm the results, in vitro enzyme inhibition assays must be performed.

**Figure 3:**
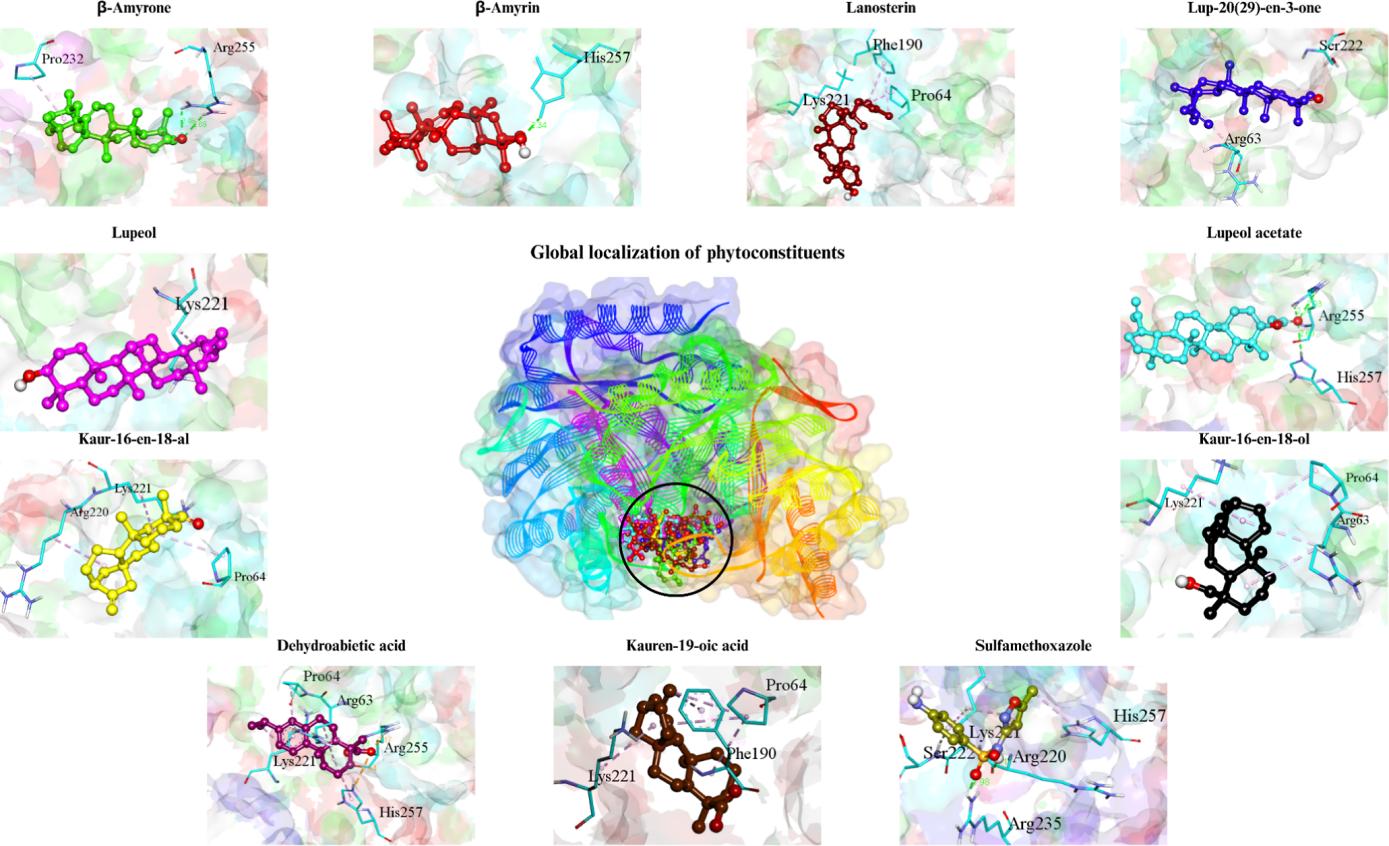
Target–ligand complex between the DHPS enzyme and the molecular structures of the phytoconstituents of *S. depilis* and *F. varia* and the enzyme inhibitor.

**Figure 4:**
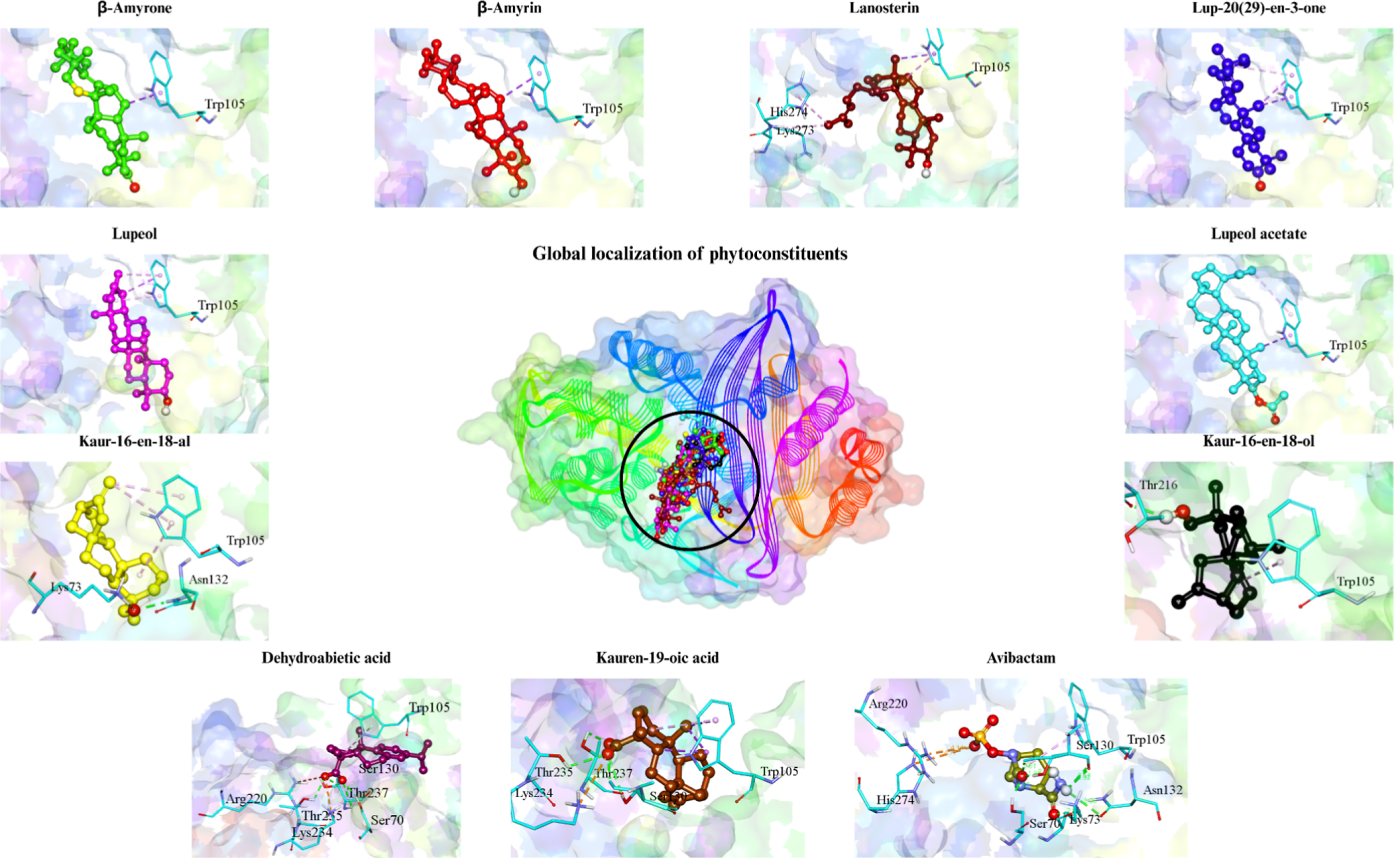
Target–ligand complex between the KPC-2 enzyme and the molecular structures of the phytoconstituents of *S. depilis* and *F. varia* and the enzyme inhibitor.

**Table 4:** Binding Affinities and Intermolecular Interactions of the Main Phytoconstituents Identified in the Extract of *S. depilis* (SD) and *F. varia* (FV) and Inhibitors of the Enzymes DHPS (IN1) and KPC-2 (IN2)

		DHPS				KPC-2			
WFE	compound	affinity (kcal/mol)	residue	interaction	distance (Å)	affinity (kcal/mol)	residue	interaction	distance (Å)
SD	β-Amyrone	–9.1	Pro232	alkyl	4.20	–9.1	Trp105	Pi-Sigma	3.42
			Arg255	hydrogen bond	2.46 and 2.69				
	β-Amyrin	–9.1	His257	hydrogen bond	2.34	–9.3	Trp105	Pi-Sigma	3.99
	Lanosterin	–7.9	Pro64	alkyl	4.64	–8.6	Trp105	Pi-Sigma	3.65 and 3.88
			Phe190	Pi-Alkyl	4.75 and 5.41		Lys273	alkyl	5.09
			Lys221	alkyl	4.94		His274	Pi-Alkyl	4.73
	Lup-20(29)-en-3-one	–8.8	Arg63	alkyl	4.83	–9.3	Trp105	Pi-Sigma	3.43 and 3.96
			Ser222	carbon hydrogen	3.34			Pi-Alkyl	5.36
	Lupeol	–8.0	Lys221	alkyl	4.62	–9.9	Trp105	Pi-Sigma	3.82
								Pi-Alkyl	3.95 and 4.43
	Lupeol acetate	–8.6	His257	hydrogen bond	2.60	–10.2	Trp105	Pi-Sigma	3.67
			Arg255	hydrogen bond	2.35 and 2.93			Pi-Alkyl	5.24
FV	Kaur-16-en-18-al	–7.2	Pro64	alkyl	5.18	–8.5	Lys73	hydrogen bond	2.67
			Arg220	alkyl	5.31		Trp105	Pi-Alkyl	4.51, 4.66, and 5.13
			Lys221	alkyl	5.36		Asn132	hydrogen bond	2.19
	Kaur-16-en-18-ol	–6.6	Arg63	alkyl	4.98 and 5.18	–7.2	Trp105	Pi-Alkyl	5.08
			Pro64	alkyl	5.47		Thr216	hydrogen bond	2.01
			Lys221	alkyl	5.19				
	Dehydroabietic acid	–7.5	Arg63	alkyl	4.95	–9.0	Ser70	hydrogen bond	2.18
			Pro64	Pi-Alkyl	5.35		Trp105	Pi-Alkyl	5.30
			Lys221	alkyl	3.45		Ser130	hydrogen bond	2.39
				Pi-Alkyl	4.45		Arg220	attractive charge	5.55
			Arg255	salt bridge and attractive charge	2.17		Lys234	attractive charge	4.55
				hydrogen bond	2.18		Thr235	hydrogen bond	2.49
			His257	attractive charge	3.94		Thr237	hydrogen bond	2.95
				Pi-Alkyl	5.08				
	Kauren-19-oic acid	–7.5	Pro64	alkyl	4.49, 4.84, and 5.40	–8.6	Trp105	Pi-Sigma	3.71, 3.79, and 3.87
			Phe190	Pi-Alkyl	4.27			Pi-Alkyl	5.40
			Lys221	alkyl	4.29		Ser130	hydrogen bond	2.06 and 2.82
							Lys234	attractive charge	4.41
							Thr235	hydrogen bond	2.70
							Thr237	hydrogen bond	2.40
IN1	Sulfamethoxazole	–6.5	Arg220	hydrogen bond	2.17	-			
			Lys221	Pi-Alkyl	4.45 and 5.06				
			Ser222	Pi-donor hydrogen bond	3.19				
			Arg235	hydrogen bond	1.98				
			His257	Pi-Alkyl	4.71				
IN2	Avibactam	-ondLys73Trp105Pi-AlkylSer130hydrogen bondAsn132hydrogen bondArg220attractive chargeHis274attractive charge					Ser70	hydrogen bond	1.82
							Lys73	hydrogen bond	2.82
							Trp105	Pi-Alkyl	4.70
							Ser130	hydrogen bond	1.91, 2.52, 2.57, and 2.61
							Asn132	hydrogen bond	3.06
							Arg220	attractive charge	4.14
							His274	attractive charge	4.77

Among the compounds tested for DHPS, β-amyrin, β-amyrin, lup-20(29)-en-3-one, and lupeol acetate stood out for having the lowest binding energies (−9.1 to −8.6 kcal/mol), surpassing the reference inhibitor sulfamethoxazole (−6.5 kcal/mol). The active site of the enzyme is composed of the amino acids Thr62, Arg63, Pro64, Gly189, Phe190, Lys221, Ser222, and Arg255.^14^

The β-amyrin compound formed two hydrogen bonds with Arg255 (2.46 and 2.69 Å), which may favor complex stabilization, especially since it involves an amino acid belonging to the active site. β-Amyrin formed a hydrogen bond with His257, which, although not directly inserted into the active site, may contribute to the predicted binding affinity (−9.1 kcal/mol). Similarly, lup-20(29)-en-3-one interacted with Arg63 (4.83 Å) and Ser222 (3.34 Å), both directly associated with the enzyme’s active site. Lupeol acetate, in turn, established hydrogen bonds with Arg255 (2.35 and 2.93 Å), in addition to additional hydrophobic interactions (π-alkyl) around 5 Å, suggesting favorable accommodation within the binding pocket.

The recurring presence of alkyl and π–alkyl interactions at short distances (2.3–4.5 Å) indicates that these compounds fit efficiently into the hydrophobic cavity of DHPS, contributing to the stabilization of the predicted complexes. Taken together, these interactions suggest a potential molecular basis for DHPS binding; however, they should be interpreted as theoretical predictions rather than direct evidence of enzymatic inhibition.

Regarding the KPC-2 enzyme, the main amino acid residues involved in its inhibition include Ser130, Lys234, Trp105, Thr235, and Thr237.^15^ The compounds analyzed showed binding energies even more favorable than those observed for DHPS, ranging from −7.2 to −10.2 kcal/mol. The best performances were recorded for lupeol acetate (−10.2 kcal/mol), lupeol (−9.9 kcal/mol), β-amyrin, and lup-20(29)-en-3-one (−9.3 kcal/mol), all surpassing the reference inhibitor avibactam (−8.3 kcal/mol).

The main interactions occurred with residues Trp105, Ser130, Lys234, Thr235, and Thr237, all located at the active site of KPC-2. The predominant types of interactions were π–sigma, π–alkyl, and hydrogen bonds, with short distances (1.9–3.9 Å), which are commonly associated with stable docking poses in silico. Lupeol acetate stood out for forming a π–sigma interaction with Trp105 (3.67 Å) and additional hydrogen bonds, indicating favorable positioning within the catalytic region of the enzyme.

Among the compounds of *F. varia*, dehydroabietic acid (−9.0 kcal/mol) and kauren-19-oic acid (−8.6 kcal/mol) formed multiple hydrogen bonds with Ser130, Lys234, Thr235, and Thr237, residues essential for β-lactam ring hydrolysis, indicating direct interaction with the active site.^15^ These interactions suggest possible interference with the active site architecture, although functional inhibition cannot be inferred solely from docking results.

Overall, the results show that *S. depilis* triterpenes exhibited higher binding affinities and direct interactions with residues of the active site in both enzymes, especially Trp105, Ser130, Lys234, Thr235, and Thr237 in KPC-2 and Arg63, Ser222, and Arg255 in DHPS. These findings support a plausible molecular interaction framework that may help explain the observed antimicrobial activity; however, the results should be interpreted as hypothesis-generating and require further experimental validation, such as enzymatic inhibition assays, to confirm the proposed mechanisms of action.

### Antimicrobial Assay

The antibacterial activity of the ethanolic extracts was confirmed by the microdilution method, showing activity against all evaluated microorganisms, with MIC values ranging from 32 to 4096 μg/mL (Table 5). The antibacterial effect of propolis was greater against Gram-positive bacteria than against Gram-negative microorganisms. This can be explained by specific features of Gram-negative bacteria, such as the structure of the cell wall, which includes an outer membrane, as well as the production of hydrolytic enzymes that degrade the bioactive molecules of propolis.^32^

**Table 5:** MIC and MBC Values of WFE-FV and WFE-SD against the Tested Microorganisms

	MIC/MBC (μg/mL)	
microorganism	WFE-FV	WFE-SD
*S. aureus* ATCC 25923	32/32	2048/4096
*S. aureus* ATCC 33591	32/32	32/32
*E. coli* ATCC 25922	512/512	512/512
*P. aeruginosa* ATCC 27853	512/512	512/512
*P. aeruginosa* PA01	512/512	512/512
*K. pneumoniae* ATCC 70063	64/128	32/32
*C. albicans* ATCC 10231	64/128	64/64
*C. parapsilosis* LFBM 03	128/256	128/256

The results obtained with propolis produced by bees of the genus *Frieseomelitta* are consistent with those from other studies that determined the MIC of propolis extracts. Souza et al. confirmed antibacterial activity against *S. aureus* (MIC 125 μg/mL), *E. coli* (MIC 125 μg/mL), and *P. aeruginosa* (MIC 62.5 μg/mL).^24^ Rocha et al. reported antibacterial activity of hydroethanolic extracts against *E. coli* with an MIC of 1024 μg/mL.^33^ Isidorov et al., in a study with propolis produced by bees of the genus *Scaptotrigona*, described similar results against *S. aureus*, *E. coli*, and *P. aeruginosa*, with MICs of 124 μg/mL, 500 μg/mL, and 500 μg/mL, respectively.^34^ However, Surek et al., in a study with ethanolic extracts of *Scaptotrigona* spp., reported MICs higher than 1000 μg/mL against *E. coli*, *K. pneumoniae*, and *P. aeruginosa*.^35^

The antibacterial property of propolis is well described; this product can increase bacterial cell membrane permeability, inhibit ATP production and bacterial motility, disrupt membrane potential, and reduce bacterial RNA and DNA synthesis.^36,37^

Therefore, the diversity and concentration of bioactive compounds represent an advantage of propolis as an antibacterial agent, since this complexity hinders the development of bacterial resistance.^36^ However, its effectiveness depends on the chemical composition of the product, which is directly influenced by climatic factors, collection period and method, and variability of plant species.^38^ The antibacterial effect of propolis has been associated with phenolic compounds, such as flavonoids, phenolcarboxylic and hydroxycinnamic acids and their esters, as well as terpenoids.^39,40^ Extracts with higher diterpene content exhibit MIC values up to twice those of extracts rich in phenols and triterpenes.^34^

The results show that the relationship among metabolite quantity, docking binding affinity, and antimicrobial activity is not direct. The *S. depilis* extract had the highest number of triterpenes, and these compounds had the best binding affinities for both DHPS and KPC-2. However, this was not reflected in better antimicrobial activity, since its MIC was higher for *S. aureus* (2048 μg/mL). On the other hand, *F. varia*, even though it had fewer compounds with high interaction affinity, showed better antimicrobial effect, with an MIC of 32 μg/mL. Thus, the data indicate that it is not the quantity of metabolites identified, nor just the strength of interaction in docking, that determines antimicrobial activity, but rather the chemical profile as a whole, possible synergies between compounds, and how they actually act on the bacterial cell.

The results presented in this study demonstrate that *S. depilis* and *F. varia* produce geopropolis with distinct chemical and physical profiles. Although three colonies per species were sampled, the pooling strategy prevented evaluation of intercolony chemical variability. Therefore, future investigations incorporating independent colony-level replicates are warranted to evaluate intraspecific variation and improve the robustness of comparative analyses. Phytochemical and GC–MS analyses confirmed the presence of phenolic compounds, flavonoids, terpenoids, sterols, and other secondary metabolites, highlighting significant differences between the two species. Both extracts exhibited antimicrobial activity and were more effective against Gram-positive bacteria. These findings reinforce the potential of native stingless bee geopropolis as a natural source of bioactive compounds with pharmacological and therapeutic relevance while also highlighting the influence of the producing species, collection period, and local flora on the chemical composition and biological activity of the product.

The results of in vitro antimicrobial activity, although demonstrating an effect, did not corroborate the predictions of molecular docking. Thus, the in silico approach estimates the binding affinity between molecules and a specific target based on structural and energetic parameters, configuring a theoretical prediction.

However, biological activity depends on several physicochemical and pharmacokinetic factors, such as solubility, stability, and permeability, that are not fully addressed in computer simulations, which may explain the lack of correlation. In addition, even though they were predominant, the compounds evaluated were associated with other constituents that may exert antagonistic effects and reduce the observed potency.

As a perspective, we propose the isolation of the major compounds and re-evaluation of in vitro activity in order to experimentally validate the in silico predictions.

## References

[1] Zulhendri F., Chandrasekaran K., Kowacz M., Ravalia M., Kripal K., Fearnley J., Perera C. (2021). Antiviral, Antibacterial, Antifungal,
and Antiparasitic Properties of Propolis: A Review. Foods.

[2] Dezmirean D., Paşca C., Moise A., Bobiş O. (2021). Plant Sources
Responsible for the Chemical Composition and Main Bioactive Properties
of Poplar-Type Propolis. Plants.

[3] Shanahan M., Spivak M. (2021). Resin Use by Stingless Bees: A Review. Insects.

[4] Zulhendri F., Lesmana R., Tandean S., Christoper A., Chandrasekaran K., Irsyam I., Suwantika A., Abdulah R., Wathoni N. (2022). Recent Update on the Anti-Inflammatory
Activities of Propolis. Molecules.

[5] Brodkiewicz Y., Marcinkevicius K., Reynoso M., Salomon V., Maldonado L., Vera N. (2018). Studies of the Biological and Therapeutic
Effects of Argentine Stingless
Bee Propolis. J. Drug Delivery Ther..

[6] Ferreira J., Fernandes-Silva C., Salatino A., Message D., Negri G. (2017). Antioxidant
Activity of a Geopropolis from Northeast Brazil: Chemical Characterization
and Likely Botanical Origin. Evidence-Based
Complementary Altern. Med..

[7] Freitas, B. ; Pereira, J. ; Cavalcante, M. ; Alves, J. ; Felix, J. ; Mascenas, V. ; Silva, S. ; Lima-Verde, L. Lista de Abelhas do Ceará. https://www.sema.ce.gov.br/fauna-do-ceara/invertebrados/abelhas. (accessed Dec 12, 2025).

[8] Rocha V., Portela R., Anjos J., Souza C., Umsza-Guez M. (2023). Stingless
bee propolis: composition, biological activities and its applications
in the food industry. Food Prod., Process. Nutr..

[9] Rebelo, K. S. ; Yamaguchi, K. ; Maróstica, M. Chemical Composition and Therapeutic Properties of Geopropolis and Propolis of Stingless Bees from Brazil: A Review. Stingless Bee Nest Cerumen and Propolis; Springer, Cham, 2024; Vol. 2; pp 217–229.

[10] Pereira F., Barboza J., Vasconcelos C., Lopes A., Ribeiro M. (2021). Use of Stingless
Bee Propolis and Geopropolis against Cancer—A Literature Review
of Preclinical Studies. Pharmaceuticals.

[11] Woisky R., Salatino A. (1998). Analysis of propolis: some parameters and procedures
for chemical quality control. J. Apicult. Res..

[12] Harborne, A. Phytochemical methods: a guide to modern techniques of plant analysis, 3rd ed.; Springer Science & Business Media, 1998.

[13] Asem N., Abdul Gapar N. A., Abd Hapit N. H., Omar E. A., Hussaini N., Omar E. (2019). Correlation between total phenolic and flavonoid contents with antioxidant
activity of Malaysian stingless bee propolis extract. J. Apic. Res..

[14] Sheikh K., Mir R., Dar M., Wali A., Qadir I., Nazir S., Zargar M., Talath S., Sridhar S., Shareef J., Masoodi M. (2025). Phytochemical screening,
antioxidant, and antimicrobial
analysis of *Portulaca oleracea* seeds with in-silico
molecular docking insights. J. Genet. Eng. Biotechnol..

[15] Oselusi S., Sibuyi N., Meyer M., Madiehe A. (2023). Ehretia Species Phytoconstituents
as Potential Lead Compounds against *Klebsiella pneumoniae* Carbapenemase: A Computational Approach. BioMed
Res. Int..

[16] Trott O., Olson A. (2009). AutoDock Vina: Improving the Speed
and Accuracy of Docking with a
New Scoring function, Efficient optimization, and Multithreading. J. Comput. Chem..

[17] Clinical and Laboratory Standards Institute M100: Performance Standards for Antimicrobial Susceptibility Testing, 30 ed.; CLSI: Wayne, PA, 2020.

[18] Pobiega K., Kraśniewska K., Derewiaka D., Gniewosz M. (2019). Comparison of the antimicrobial
activity of propolis extracts obtained by means of various extraction
methods. J. Food Sci. Technol..

[19] Cardinal L., Tonet G., Oliveira A., Fernandes T., Bistaff A., Schleder E., Santos K., Matias R. (2022). Identidade
e Qualidade da Própolis Proveniente de Duas Regiões
do Cerrado Sul-Mato-Grossense. Ensaios Ciência.

[20] Sette K. M., Garcia A. R., Tinoco L. W., Pinheiro A. S., Rodrigues I. A. (2025). Meliponini
Geopropolis Extracts Induce ROS Production and Death in Leishmania
amazonensis Promastigotes and Axenic Amastigotes In Vitro. Biology.

[21] Villacrés-Granda I., Coello D., Proaño A., Ballesteros I., Roubik D. W., Jijón G., Granda-Albuja G., Granda-Albuja S., Abreu-Naranjo R., Maza F., Tejera E., González-Paramás A. M., Bullón P., Alvarez-Suarez J. M. (2021). Honey quality parameters, chemical
composition and
antimicrobial activity in twelve Ecuadorian stingless bees (Apidae:
Apinae: Meliponini) tested against multiresistant human pathogens. LWT.

[22] Bergamini A., de Almeida Bergamini B. V. S., Pessoa I., de Sousa
Cutrim T. A., dos Santos T. C., dos Santos M. C., da Rocha Fonseca V., Romão W., Scherer R., Endringer D., Fronza M. (2025). Chemical profile, antioxidant, antifungal, and cytotoxic
activities of propolis from the stingless bee *Tetragona clavipes*. Braz. J. Microbiol..

[23] Pazin W., Monaco L., Egea Soares A. E., Miguel F., Berretta A., Ito A. (2017). Antioxidant activities
of three stingless bee propolis and green
propolis types. J. Apic. Res..

[24] Soares, A. ; Bilezikdjian, P. ; Elias, P. ; Medeiros, P. ; Souza, L. Identidade e qualidade de diferentes extratos de própolis: Revista Gestão em Foco, 2017.

[25] Silveira Z., Macedo N., Dantas D., Brito S., Santos H., Gomes R., Coutinho H., Cunha F., Silva M. (2024). Chemical Profile
and Biological Potential of *Scaptotrigona* Bee Products
(Hymenoptera, Apidae, Meliponini): An Review. Chem. Biodiversity.

[26] Vit, P. ; Pedro, S. R. M. ; Roubik, D. W. Pot-Honey: A Legacy of Stingless Bees; Springer: New York, 2013.

[27] Souza E., Silva E., Cordeiro H., Lage-Filho N., Silva F., Reis D., Porto C., Pilau E., Costa L., Souza A., Menezes C., Flach A. (2018). Chemical Compositions
and Antioxidant and Antimicrobial Activities of Propolis Produced
By *Frieseomelitta longipes* and *Apis mellifera* BEES. Quim. Nova.

[28] Jana K., Ghosh A., Debnath B., Das S. (2023). GC-MS Analysis of Phytocomponents
of Methanolic Bark Extract of Sterculia foetida. Res. J. Pharm. Technol..

[29] Pimentel L., Teixeira F., Soares A., Costa P., Fontes A. L., Vidigal S., Pintado M. (2025). Biocompatibility, Anti-inflammatory,
and Antimicrobial Properties of Kaurenoic Acid Recovered from Synthetic
Biology By-Products: A Sustainable Approach to Food Bioactives. Waste Biomass Valorization.

[30] Hao M., Xu J., Wen H., Du J., Zhang S., Lv M., Xu H. (2022). Recent Advances on
Biological Activities and Structural Modifications
of Dehydroabietic Acid. Toxins.

[31] Castro-Alvarez A., Costa A., Vilarrasa J. (2017). The Performance of Several Docking
Programs at Reproducing Protein–Macrolide-Like Crystal Structures. Molecules.

[32] Sforcin J. (2016). Biological
Properties and Therapeutic Applications of Propolis. Phytother. Res..

[33] Rocha V., Portela R., Lacerda L., Sokolonski A., de Souza C. O., dos Anjos J. P., Nascimento R., Umsza-Guez M. (2023). Propolis from different Brazilian stingless bee species:
phenolic composition and antimicrobial activity. Food Prod., Process. Nutr..

[34] Isidorov V., Maslowiecka J., Szoka Ł., Pellizzer N., Miranda D., Olchowik-Grabarek E., Zambrzycka M., Swiecicka I. (2022). Chemical Composition and Biological
Activity of Argentinian
Propolis of Four Species of Stingless Bees. Molecules.

[35] Surek M., Fachi M., Cobre A., Oliveira F., Pontarolo R., Crisma A., Souza W., Felipe K. (2021). Chemical composition,
cytotoxicity, and antibacterial activity of propolis from Africanized
honeybees and three different Meliponini species. J. Ethnopharmacol..

[36] Przybyłek I., Karpiński T. (2019). Antibacterial
Properties of Propolis. Molecules.

[37] Almuhayawi M. (2020). Propolis as
a novel antibacterial agent. Saudi J. Biol.
Sci..

[38] Tsadila C., Amoroso C., Mossialos D. (2023). Microbial Diversity in Bee Species
and Bee Products: Pseudomonads Contribution to Bee Well-Being and
the Biological Activity Exerted by Honey Bee Products: A Narrative
Review. Diversity.

[39] Patricio E., Cruz-Lopez L., Maile R., Tentschert J., Jones G., Morgan E. (2002). he propolis of stingless bees: terpenes
from the tibia of three *Frieseomelitta* species. J. Insect Physiol..

[40] Salatino A. (2022). Perspectives
for Uses of Propolis in Therapy against Infectious Diseases. Molecules.

